# Drug strategies for the treatment and prevention of proliferative vitreoretinopathy: an overview of innovative treatment concepts

**DOI:** 10.1007/s10792-026-03963-6

**Published:** 2026-01-31

**Authors:** Adrian Konstantin Luyken, Valentin Junge, André Schulz, Thomas Armin Fuchsluger, Friederike Schaub

**Affiliations:** Department of Ophthalmology, Rostock University Medical Center, Doberaner Str. 140, 18057 Rostock, Germany

**Keywords:** Proliferative vitreoretinopathy, Retinal detachment, Epithelial-mesenchymal transition, TGF-β signaling pathway, 5-fluorouracil, Methotrexate

## Abstract

**Purpose:**

To present an overview of emerging pharmacological strategies for the prevention and treatment of proliferative vitreoretinopathy (PVR).

**Methods:**

This review critically examines recent experimental and clinical evidence on pharmacological agents targeting key pathogenic mechanisms of PVR, including epithelial–mesenchymal transition, profibrotic cytokine signaling (TGF-β, PDGF, VEGF), and inflammation-driven tissue remodeling. Investigated compounds include clinically tested substances such as daunorubicin, 5-fluorouracil, corticosteroids, anti-VEGF agents, methotrexate, isotretinoin, decorin and infliximab, as well as newer experimental approaches including Topotecan, Melphalan, ROCK-Inhibitors and gene-regulated therapies. Mechanistic insights into receptor crosstalk, intracellular signaling cascades, and cell survival pathways are integrated with findings from preclinical models and clinical studies.

**Results:**

Several agents have shown anti-proliferative and anti-inflammatory effects in vitro and in vivo, with methotrexate and infliximab emerging as particularly promising candidates. However, clinical data remain heterogeneous, and no pharmacological agent has yet received regulatory approval for PVR treatment. Risk stratification based on preoperative PVR, vitreous hemorrhage, or ocular trauma may help optimize patient selection in future trials.

**Conclusion:**

Pharmacological modulation of PVR is conceptually well supported by preclinical data, but clinical translation remains limited. Well-designed randomized trials in clearly defined high-risk populations are needed to validate efficacy, determine optimal treatment windows, and develop standardized protocols for both prophylaxis and therapy.

## Introduction

**Fig. 1:**
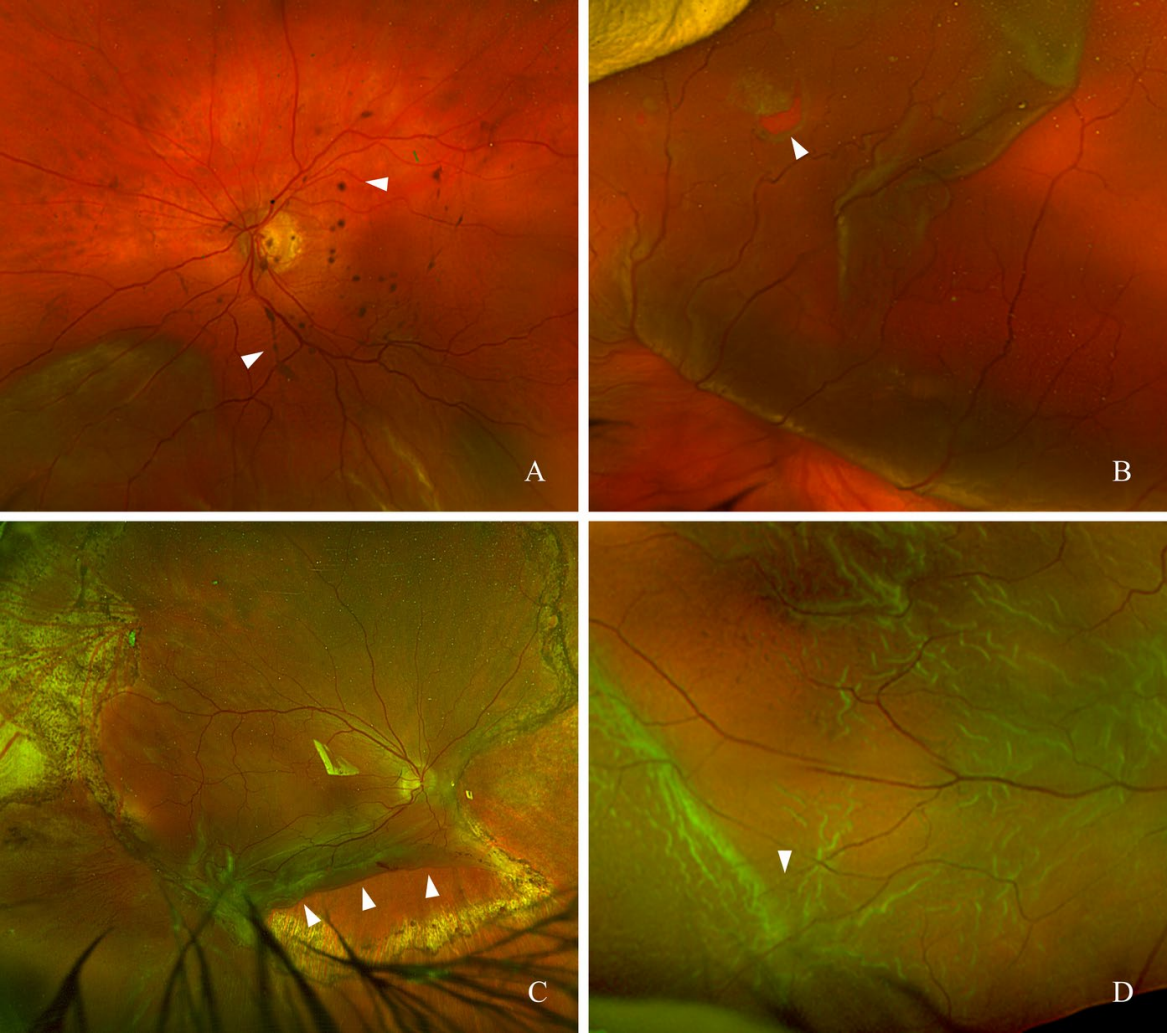
**A** PVR grade A-B with pigment in the vitreous cavity (arrows), **B** PVR grade B with rolled hole edge (arrow), **C** PVR grade C with rolled in retinal flap after relaxing retinectomy (arrows), **D** PVR grade C with star-shaped folds (arrow)

Proliferative vitreoretinopathy (PVR) is one of the most serious complications following rhegmatogenous retinal detachment (RRD) and trauma involving the posterior segment of the eye. Representing a dysregulated wound-healing response, PVR develops in approximately 5–10% of cases after surgical intervention for primary RRD and in 25–50% of cases following recurrent surgery for re-detachment [1, 2]. Following the updated Retina Society Classification PVR severity is divided into grades A, B, and C, with grade C defined by full-thickness retinal folds (Fig. 1). This updated classification provides a more detailed description of anterior and posterior PVR [3]. A distinction is made between post-traumatic and postoperative forms. Traction generated by fibrocellular, epiretinal, and subretinal membranes can lead to secondary retinal breaks and is the primary cause of recurrent retinal detachment after initially successful repair [4]. Despite advances in surgical techniques, in recent years, anatomical success rates in eyes with established PVR have remained unchanged at approximately 75%, highlighting the ongoing need for effective pharmacological prophylaxis and treatment strategies [5]. Although numerous preclinical and clinical studies have evaluated pharmacological interventions for PVR, until today none have been translated into standard clinical care. The gap between experimental efficacy and clinical applicability remains a central challenge in the development of pharmacological PVR therapies [6].

### Aims of this review

Because primary RD, recurrent RD and trauma carry distinct PVR risks, and because early PVR prevention and treatment of established PVR involve different biological processes and therapeutic targets, these conditions must be evaluated separately rather than treated as a single uniform disease entity. This review mainly focuses on PVR prophylaxis and therapy in primary RRD. All extracted study characteristics and outcomes are summarized in Table 1.

**Table 1:** Summary of key studies on primary prevention of proliferative vitreoretinopathy (PVR) following rhegmatogenous retinal detachment (RRD)

Substance	Drug class	Mechanism of action	Preclinical data	Clinical study	Study Type and Number of cases	Condition	Primary endpoint	Main findings	Safety	Comment/status
MTX	Folic acid antagonist	Inhibits DNA synthesis (DHFR) and inflammatory cytokines	RPE and PVR membrane models; animal studies	Nourinia et al., 2024	RCT; (*n* = 74)	RRD with PVR-C	Reattachment rate at 6 months	Reduced PVR rate; no significant effect on the reattachment rate	No relevant safety risks	Clinically promising; further Trials needed
Daunorubicin	Anthracycline antibiotic	Inhibits topoisomerase II and cell proliferation	RPE and glial cell models; animal studies	Wiedemann et al., 1998	RCT; (*n* = 286)	RRD with PVR-C	Reattachment rate at 6 months	Reduced reoperation rate; tendency toward increased reattachment rate	No relevant safety risks	Limited evidence; not routinely used, further Trials needed
5-FU + LMWH	Antimetabolite + anticoagulant	Inhibits cell proliferation and ECM remodeling	RPE, fibroblast models; animal studies	PRIVENT Trial (Schaub et al., 2022)	RCT; (*n* = 325)	RRD with high risk for PVR	PVR rate	No effect	No relevant safety risks	No proven benefit; not recommended
Infliximab	Anti-TNF-α monoclonal antibody	Inhibits TNF-α–mediated inflammation and EMT	In vitro inflammation and EMT models; animal data	FIXER Trial (Younes et al., 2024)	RCT; (*n* = 60)	RRD with PVR-C	Reattachment rate at 6 months	Reduced reoperation rate; tendency toward increased reattachment rate	No relevant safety risks	Preliminary efficacy; further trials needed
Isotretinoin	Retinoid	Inhibits EMT via RAR-β and TGF-β/CTGF pathways	RPE models; rabbit PVR model	DELIVER Trial (London et al., 2019)	Randomized open label, dual-cohort study with pathology matched historical controls; (*n* = 84)	RRD with high risk for PVR and recurrent RRD due to PVR	Reattachment rate at 3 months	No effect on reattachment rate in recurrent RD, increased reattachment rate in primary RD	No relevant safety risks, high number of mild side effects (dry skin and mucus membranes)	Systemic side effects limit applicability (dry skin and mucus membranes)
Decorin	Endogenous proteoglycan	Inhibits TGF-β signaling and fibrosis	Fibrosis models in multiple organs; rabbit PVR model	Abdullatif et al., 2023	Prospective pilot study; (*n* = 12)	Perforating globe injury	ERG a and b implicit time and latency at 3 months	No toxicity; no progression in 75% of PVR scars	No relevant safety risks	Early clinical data; further validation required
Anti-VEGF	VEGF inhibitor	Inhibits VEGF signaling and PDGFRα activation	Limited in vitro and cytokine data	Tousi et al., 2016	Randomized open label; (*n* = 27)	RRD with high risk for PVR	Reattachment rate at 6 months	No anatomical or functional benefit	No relevant safety risks	No proven benefit; not recommended
Corticosteroids	Slow release Dexamethason	Inhibits inflammatory cytokines and cell migration	RPE/cytokine models; trauma model	Banerjee et al., 2017	RCT; (*n* = 140)	RRD with PVR-C	Reattachment rate at 6 months	No anatomical benefit, reduction of CMO	No relevant safety risks	No proven benefit; not recommended
	Triamcinolon		RPE/cytokine models; trauma model	ASCOT Trial (Casswell et al., 2024)	RCT; (*n* = 280)	Perforating globe injury	Visual Acuity Improvement	No anatomical or functional benefit	No relevant safety risks	No proven benefit; not recommended
Melphalan	Topoisomerase I inhibitor	Inhibits DNA replication and repair	RPE and fibroblast models; rabbit trauma model	Moreira-Neto et al., 2025	Prospective pilot study; (*n* = 6)	RRD with ˃PVR-CP2	Ocular Toxicity, PVR rate at 3 months	No toxicity; no PVR recurrence	No relevant safety risks	Early clinical data; further validation required
Topotecan	Alkylating cytostatic agent	Cross-links DNA and induces apoptosis	Rabbit PVR model (24 eyes)	TOPO-RD (NCT05523869, Phase II)	Randomized open label study (*n* = 50)	RRD with PVR-C or recurrent RRD due to PVR	Reattachment rate at 6 months			No clinical data; currently under investigation

## Methodology

We performed a comprehensive literature search covering all available publications up to November 2025. Searches were conducted in Medline, PubMed, the Science Citation Index (Web of Science), and the Cochrane Library. Keywords related to rhegmatogenous retinal detachment and proliferative vitreoretinopathy were combined with terms for medical therapies, including methotrexate, corticosteroids, anti-VEGF agents, and antiproliferative or antineoplastic drugs. Clinical trial registries (e.g., ClinicalTrials.gov) were additionally screened. Only English-language publications were considered. Titles, abstracts, and full texts were assessed for relevance to the scope of this review, and reference lists of included studies were screened to identify additional pertinent sources.

### Pathophysiology of PVR

PVR develops after an initial retinal injury triggering a dysregulated wound healing response. Retinal pigment epithelial (RPE) cells migrate into the vitreous cavity and undergo epithelial–mesenchymal transition (EMT).

In parallel, pro-inflammatory mediators such as interleukin-6 (IL-6) and monocyte chemoattractant protein-1 (MCP-1) recruit macrophages. These cells release reactive oxygen species (ROS), which indirectly activate PDGFRα and amplify profibrotic signaling [7]. These pathways promote proliferation, extracellular matrix (ECM) production, and cellular contractility [8]. The resulting membranes consist of myofibroblasts, collagen types I and III, and fibronectin, contributing to tractional forces and secondary retinal tears [9].

### Cellular mechanisms of membrane formation

Key cellular targets for pharmacological intervention include:

- **RPE cell activation**: Microenvironmental changes after retinal injury, characterized by increased cytokines, growth factors, ECM remodeling and loss of epithelial polarity, collectively trigger dislocated RPE cells to transition into a mesenchymal state [9, 10].
- **PDGFRα signaling**: PDGFRα can be activated independently of PDGF by non-canonical ligands such as HGF and EGF, via Src kinases and ROS. This leads to sustained survival signaling [11].
- **VEGF–PDGF crosstalk**: VEGF competes with PDGF for PDGFRα binding, increasing the number of unbound receptors. These receptors are susceptible to activation by alternative ligands, which enhance PI3K/Akt signaling and suppress p53-mediated apoptosis [12, 13].
- **Cells in PVR Membranes:** Hyalocytes and glial cells act as upstream inflammatory and fibrotic amplifiers, driving the cellular recruitment, cytokine signaling and myofibroblast formation that underlie PVR [14, 15].

### Therapeutic targets

#### EMT

Displaced RPE are exposed to various cytokines and growth factors, which induce their transition into mesenchymal-like cells [16]. Transforming TGF-β, acting as a central regulator, promotes the expression of α-SMA and collagen type I via activation of the Smad2 and pTAK1/p38 signaling pathways. This process results in the loss of epithelial polarity and acquisition of contractile properties, enabling RPE cells to contribute to fibrocellular membrane formation [16].

#### VEGF–PDGF signaling interactions

Crosstalk between VEGF and PDGF signaling pathways also significantly contributes to PVR development. VEGF interferes with PDGF binding to PDGFRα, leading to an accumulation of unbound receptor monomers. These receptors become susceptible to indirect activation by alternative ligands, initiating downstream PI3K/Akt signaling and thus suppressing p53 and thereby supporting cell survival and inhibiting apoptosis. Elevated intraocular VEGF concentrations in eyes with PVR and within epiretinal membranes further underscore the relevance of this signaling axis as a potential therapeutic target [17].

#### Cells in PVR membranes and the role of the vitreous body

PVR membranes in primary RRD contain a mixed population of cells, including RPE cells, myofibroblasts, macrophages, glial cells, hyalocytes and additional myeloid and progenitor cell types [14].

Adjacent to RPE cells, fibroblast-like cells can also differentiate into myofibroblasts under the influence of TGF-β and Rho signaling, and both fibroblasts and myofibroblasts contribute contractile forces within epiretinal membranes [18]. Hyalocytes and glial cells in the vitreous are also non-negligible drivers of PVR and represent relevant therapeutic targets. The concept of Vitreoschisis with persistent vitreous cortex remnants (VCR) provides a substrate that retains and activates these cells [19, 20]. Incomplete posterior vitreous detachment (PVD) leaves cortical lamellae adherent to the retina, enabling recruitment of hyalocytes, Müller cells and microglia.

Hyalocytes on these remnants secrete proinflammatory and profibrotic mediators such as IL-6, TNF-α and TGF-β, which amplify cell migration, proliferation and matrix remodeling [21]. Glial cells reinforce this response by contributing additional cytokines and matrix components. Single-cell and transcriptomic analyses of human PVR membranes show marked overexpression of extracellular matrix proteins including FN1 and SPARC [22] and demonstrate a TGF-β2-dependent transdifferentiation of hyalocytes into α-SMA-positive myofibroblasts [23]. Histopathology confirms a progression from hyalocyte-rich lamellar collagen to fibrotic stages dominated by myofibroblasts [20]. These findings position hyalocytes and glial cells as targetable regulators of PVR pathogenesis.

#### PVR risk stratification and patient selection

Numerous approaches have been proposed to identify patients at risk for developing PVR. However, a standardized risk assessment has not been established. Early efforts, such as the risk formula by Asaria et al., aimed to quantify individual risk using clinical parameters, while more recent studies have explored objective biomarkers [24–26]. More recently, the PRIVENT study demonstrated that elevated aqueous flare values measured by laser flare photometry were associated with a significantly increased risk of postoperative PVR. A flare value of ≥ 15 photons/ms was used as the main inclusion criterion. Identifying high-risk patients remains a prerequisite for preventive treatment and is essential for targeted clinical trial design [27, 28].

A recent comprehensive meta-analysis identified preoperative PVR as the strongest predictor of postoperative disease progression (odds ratio: 22.28; 95% confidence interval: 2.54–195.31) [29]. Additional risk factors include smoking (OR: 5.07), vitreous hemorrhage (OR: 4.12), choroidal detachment (OR: 4.45), and macula-off status (OR: 1.85) [29]. Preoperative risk stratification and structured postoperative follow-up are essential to enable individualized prophylactic strategies and form the foundation for current and future therapeutic developments.

### Substances in clinical trials

The following section provides an overview of substances that have been investigated in clinical trials, as well as experimental therapeutic approaches examined in in vitro studies and animal models. In addition, overarching therapeutic principles that apply across different drug classes are discussed.

### Altered pharmacokinetics in silicone oil endotamponades

Following retinal detachment repair, the choice of endotamponade (e.g., BSS, gas, or silicone oil) critically influences the pharmacokinetics of intravitreally administered drugs and therefore plays a decisive role in the effectiveness of pharmacological PVR prophylaxis. Silicone oil, frequently used in eyes at high risk for recurrent detachment or PVR formation, profoundly alters intraocular drug behavior. Depending on a drug’s molecular weight, polarity (lipophilic vs. hydrophilic), and solubility characteristics, its half-life, compartmental distribution, and biological activity may change substantially [30–32].

Hydrophilic small molecules often accumulate within the aqueous interface fluid, resulting in high local concentrations, but are rapidly cleared from vitrectomized cavities, whereas lipophilic agents may partition into silicone oil and consequently fail to achieve therapeutic levels at the retinal surface [33].

In particular, intravitreal corticosteroids show markedly different pharmacokinetics depending on whether they are delivered into a vitrectomized eye, or an eye filled with silicone oil. In non-vitrectomized eyes, depot formulations such as triamcinolone or sustained-release dexamethasone implants remain longer due to slow diffusion through the gel vitreous. After vitrectomy, clearance accelerates and drug activity is shortened, as shown for the dexamethasone implant in diabetic macular edema [31]. In silicone-oil–filled eyes, pharmacokinetics are altered even more as in in-vitro studied demonstrated that dexamethasone implants show irregular and incomplete drug release, remain structurally intact without normal degradation, and, particularly in denser silicone oils, fail to exert anti-inflammatory effects despite measurable drug concentrations, indicating that silicone oil can markedly impair diffusion and bioavailability [32].

#### Anti-inflammatory approaches

Intraocular inflammation contributes to the pathogenesis of PVR, and corticosteroids have long been considered because of their broad anti-inflammatory and antiproliferative activity. Triamcinolone acetonide suppresses inflammatory mediators such as IL-6 and MCP-1 but shows limited efficacy as monotherapy [34]. Early preclinical PVR models demonstrated dose-dependent benefits: in a refined rabbit model, intravitreal triamcinolone at 2 mg reduced PVR-related retinal detachment from 90 to 56%, whereas higher doses provided no additional effect, indicating an optimal dose around 2 mg [35]. In a related model, simultaneous injection of triamcinolone with fibroblasts reduced detachment rates from 93 to 75%, and prophylactic administration 24 h before PVR induction decreased detachment rates from 85 to 43% [36].

These preclinical results contrast with inconsistent clinical findings. A randomized trial in eyes with RD and PVR grade C found no benefit of adjunctive intraoperative triamcinolone during vitrectomy with silicone oil [37]. Small uncontrolled series using 4 mg intravitreal triamcinolone reported contradictory functional outcomes and inconsistent suppression of reproliferation [38], and another series in advanced PVR C2 reported high reattachment rates but lacked a control arm [39].

Systemic corticosteroids have also been investigated, but oral prednisolone demonstrated weaker antifibrotic effects and no meaningful visual advantage compared with intravitreal triamcinolone [40]. Sustained-release corticosteroid systems maintain intraocular drug levels for prolonged periods [41, 42], yet a large randomized trial using a 0.7 mg dexamethasone implant in vitrectomized eyes with PVR grade C showed no anatomical or functional benefit compared with placebo [37].

In trauma-associated PVR, where the risk profile differs substantially from primary RD, evidence also remains conflicting. Early triamcinolone injection during primary repair reduced traumatic PVR severity and improved reattachment in one randomized study [43], whereas a large multicenter phase III trial evaluating intravitreal and subtenon triamcinolone during secondary vitrectomy demonstrated no benefit and worse anatomical outcomes, along with more steroid-related complications [44]. Combination therapy with heparin likewise failed to demonstrate clinical impact in established PVR [45].

Overall, although corticosteroids exhibit mechanistic activity relevant to early PVR development, current clinical evidence does not support their routine use for PVR prophylaxis after primary RD, and their effect is strongly dependent on timing, dose, route of administration, and underlying risk profile.

#### Antiproliferative strategies

##### Daunorubicin

Daunorubicin is a cytotoxic anthracycline antibiotic that inhibits topoisomerase II, thereby blocking cell division, particularly in RPE and glial cells. In one of the largest multicenter randomized trials on PVR, Wiedemann et al*.* (1998) investigated the intraoperative administration of daunorubicin (7.5 µg/mL via infusion for 10 min) in 286 eyes with established grade C PVR. Over a one-year follow-up, the reoperation rate was significantly lower in the daunorubicin group (*p* = 0.05), while the rate of primary retinal reattachment was comparable between groups. However, the study population was highly heterogeneous in terms of PVR etiology, severity, and surgical history, complicating the interpretation of results [46].

Several years later, Kumar et al*.* evaluated intravitreal injection of 5 µg daunorubicin prior to vitrectomy in patients with PVR. The treatment group showed significantly lower vitreous opacity (*p* = 0.0044) and a trend toward improved anatomical and functional outcomes at three months. The study’s conclusions were limited by small sample size and clinical heterogeneity [47]. In summary, daunorubicin has demonstrated antiproliferative and anti-inflammatory properties in both preclinical and clinical studies. A consistent advantage with regard to primary retinal reattachment has not been demonstrated. The use of daunorubicin as a purely prophylactic agent has not yet been systematically studied. Due to its favorable safety profile at tested doses, particularly the absence of retinal toxicity, it may represent a promising candidate for future controlled trials. The heterogeneity of existing studies remains a key limitation to drawing firm conclusions.

##### 5-FU and low-molecular-weight heparin (LMWH)

5-FU is an antimetabolite that inhibits DNA and RNA synthesis. LMWH has demonstrated antiproliferative effects in addition to its anticoagulant properties, including inhibition of RPE cell proliferation and modulation of pro-fibrotic cytokine activity [48, 49]. The combination has shown additive effects in vitro and was considered promising for PVR prevention. Early case series suggested a potential benefit of intraoperative application of 5-FU and LMWH in high-risk eyes undergoing retinal detachment surgery [50]. However, these findings could not be confirmed in a large, randomized, double-blind, placebo-controlled multicenter trial including 325 high-risk patients with primary RRD. Risk stratification was performed by laser flare photometry. Intraoperative infusion of 5-FU (200 mg/mL) and dalteparin (5 IU/mL) showed no significant reduction in the rate of PVR compared to placebo (PVR: 28% vs. 23%, *p* = 0.77). Secondary outcomes, including re-detachment rates and visual acuity, also did not differ between the groups. [27]. More recently, a prospective randomized study in 42 pediatric eyes with high-risk pediatric RRD compared intraoperative infusion of 5-FU/LMWH versus placebo. Although the difference in re-detachment rate (19% vs. 33%) did not reach statistical significance, the treatment group showed later onset of recurrence (9.5 vs. 2.9 weeks, *p* = 0.042), a lower rate of advanced PVR (*p* = 0.038), and significantly better final visual acuity outcomes (*p* = 0.035) [51].

While this pediatric study suggests a possible benefit in specific subgroups, the overall clinical evidence remains inconclusive. Given the lack of efficacy in the large adult trial and the limited data in children, routine use of 5-FU and LMWH for PVR prophylaxis cannot currently be recommended outside of controlled clinical settings.

##### Isotretinoin (13-cis-retinoic acid)

Isotretinoin, a synthetic retinoid widely used in dermatology, is known to inhibit EMT in RPE cells by activation of retinoic acid receptor-β (RAR-β), reduces TGF-β1 and connective tissue growth factor (CTGF) expression, and suppresses PDGF-induced proliferation. In preclinical studies, isotretinoin reduced the incidence of PVR in a rabbit model by downregulating α-SMA and collagen I [52]. Clinical results are heterogeneous. The DELIVER trial (2023) demonstrated a significantly higher retinal reattachment rate (84.5% vs. 61.1%; *p* = 0.005) in high-risk patients after primary vitrectomy receiving 20 mg/day isotretinoin for 12 weeks, whereas no benefit was observed in recurrent cases [53]. Earlier studies suggest dose-dependent effects [54, 55]. Despite encouraging results, systemic side effects such as cheilitis and dry eye, along with lack of formal approval, currently restrict its use to off-label indications. Future studies should explore intraocular delivery to reduce systemic toxicity.

##### Methotrexate (MTX)

MTX is a folate antagonist with both immunosuppressive and cytostatic properties, depending on dosage. It inhibits cell proliferation by blocking dihydrofolate reductase and thereby suppressing DNA synthesis in actively dividing cells such as RPE cells in PVR. MTX has demonstrated significant antiproliferative effects on PVR membrane isolated from human patients and in cell culture models [55–57]. It also modulates pro-inflammatory cytokines such as IL-6 and TNF-α, addressing both the inflammatory and fibrotic components of PVR [58]. Since 2019, multiple case series and smaller trials have reported encouraging outcomes with MTX in different dosing regimens (ranging from 100 to 400 µg) and different delivery modalities including intravitreal infusions (in this case 40 mg MTX solved in 500 ml balanced salt solution (BSS) and single and repeated postoperative intravitreal injection in PVR management, with no evidence of retinal toxicity to date [59–61].

Since MTX is a small molecule (molecular weight 454.44 g/mol) with a short intravitreal half-life of approximately 6–7 h, limited tissue accumulation, and a requirement for sustained exposure to exert its antiproliferative effects, arguments can be made in favor of both application strategies [59].

Because PVR pathways are activated immediately after retinal detachment and are further amplified by vitrectomy, MTX must reach therapeutic levels as early as possible, preferably during surgery [58, 62]. In this regard, intraoperative infusion offers the advantage of establishing stable retinal tissue concentrations already during vitectomy in animal models [63, 64]. By contrast, a single intravitreal injection at end of the surgery distributes poorly in silicone-oil-filled eyes, remaining largely confined to the aqueous compartment. This results in subtherapeutic drug exposure in the superior retina and potentially unpredictable concentrations in the inferior quadrants [62]. Given that intravitreal MTX injections (400 µg) maintain therapeutic levels (0.1–2 µg/mL) for only about 72 h, maintaining effective concentrations over several days requires repeated postoperative dosing, typically every two weeks [65]. To mitigate the risks associated with repeated high-concentration spikes, such as corneal epitheliopathy due to reflux of MTX through the injection site or endophthalmitis, sustained-release delivery systems are being developed. Analogous to the approved dexamethasone implant (“Ozurdex,” Allergan, Irvine, USA) [66], novel MTX formulations, including a recently introduced liposomal MTX preparation capable of providing sustained release for up to six weeks in large animal models, have shown promising preclinical results [67, 68].

A recent systematic review included 13 studies and 360 eyes, showing higher rates of successful retinal reattachment and reduced PVR recurrence associated with adjuvant MTX treatment. Both intraoperative infusion and repeated intravitreal injections have shown potential benefits, but the varying dosing schedules make it difficult to determine the optimal treatment method [69]. Reported safety outcomes were favorable, with no serious adverse events or retinal toxicity observed. However, most available data are based on small, uncontrolled studies with heterogeneous patient populations [6, 69].

A multicenter study from 2025, investigating patients taking systemic MTX and evaluating their risk for PVR-Development following RD, suggests that systemic MTX might also achieve sufficient intraocular concentrations to meaningfully influence PVR development [70].

To date, the optimal route of administration, dosing strategy, injection frequency, and treatment duration remain undefined, and no dose-escalation studies have been conducted. Critically, systematic safety data for higher intraocular doses of MTX are lacking, underscoring the need for further investigation. Robust, well-designed randomized controlled trials are required to validate clinical efficacy and to establish evidence-based dosing protocols before MTX can be adopted for routine clinical use.

##### Infliximab

Infliximab, a monoclonal antibody targeting TNF-α, represents an anti-inflammatory approach for PVR. It inhibits TNF-α–mediated activation of RPE cells and reduces expression of adhesion molecules such as ICAM-1, thereby limiting inflammatory cell migration into the vitreous cavity. This mechanism is particularly relevant in the early phase of PVR, where TNF-α contributes to EMT and cell migration [71]. Clinical evidence for infliximab is emerging. In a randomized study from 2024 including 60 patients with grade C PVR, intravitreal infliximab administered during vitrectomy resulted in a significantly higher rate of primary retinal reattachment and fewer reoperations, with no serious adverse effects observed [72]. Although these findings are promising, long-term outcomes and efficacy in non-traumatic PVR remain uncertain. Infliximab offers a targeted biologic strategy to modulate the inflammatory cascade in PVR and should be further evaluated in larger, controlled studies.

##### Topotecan und melphalan

Melphalan and Topotecan are cytotoxic agents primarily known from oncological applications, especially in the treatment of intraocular retinoblastoma with vitreous seeding [73]. Melphalan is an alkylating agent that exerts its effect by forming DNA cross-links, thereby inducing cell cycle arrest and apoptosis in rapidly dividing cells. Topotecan is a topoisomerase I inhibitor that interferes with DNA replication and transcription by stabilizing the cleavable complex between DNA and the enzyme, leading to DNA strand breaks and cell death. Both agents have been successfully used via intra-arterial and intravitreal routes in pediatric retinoblastoma and are characterized by their potent antiproliferative activity and relative ocular tolerability when applied at controlled doses. Given their established intraocular safety profiles and strong cytotoxic effects, they are currently being investigated as potential adjuvant therapies for PVR [74]. Preclinical and pilot studies have suggested that these agents may inhibit RPE cell activity and fibroblast proliferation, based on their mechanisms of action and the known pathophysiology of PVR. However, clinical data supporting their use in this context remain limited to animal models and early-phase investigations.

### Inhibition of growth factors

#### Anti-VEGF therapy

Intravitreal VEGF inhibitors have been proposed as a potential strategy to prevent PVR. VEGF modulates the interaction between PDGF and PDGFRα at the p53 level, influencing cell proliferation and reducing apoptosis in PVR-related cells [8]. Agents such as ranibizumab not only inhibit angiogenesis but may also suppress VEGF-mediated activation of PDGFRα [8]. Existing evidence showed neither clinical benefit nor harm in terms of anatomical or functional outcomes [75, 76].

#### Decorin

Decorin is an endogenous proteoglycan that acts as a potent inhibitor of TGF-β, thereby modulating fibrotic responses through multiple pathways [77, 78]. It interferes with matrix-cell interactions, inhibits fibroblast migration, and suppresses monocyte differentiation into profibrotic phenotypes [79, 80]. Additionally, decorin regulates collagen fibril maturation, contributing to extracellular matrix stability [78]. Antifibrotic effects have been demonstrated in various organs, including the brain [81], lung [82], kidney [83], and muscle [84]. In ophthalmology, decorin has shown promising antifibrotic activity in preclinical studies. In a rabbit model of glaucoma filtration surgery, its application significantly reduced scarring without toxicity [85]. In an experimental PVR model, adjunctive decorin administration during vitrectomy decreased both the incidence and severity of PVR, with no histological signs of retinal damage [86]. The first prospective pilot study in humans, conducted by Abdullatif et al*.*, investigated decorin in twelve patients with perforating ocular trauma [87]. A single intravitreal injection (200–400 µg) was administered immediately after injury and prior to vitrectomy. The treatment was well tolerated, with no systemic or ocular adverse events. Retinal function remained stable or improved, as assessed by electroretinography, and OCT showed preserved retinal architecture. The primary reattachment rate was 75%, the PVR rate 25%, and globe retention was achieved in 87.5% of cases. In 75% of eyes, the initial PVR scar did not progress. These findings indicate that decorin, due to its broad antifibrotic activity and favorable safety profile, represents a promising candidate for PVR prophylaxis. Further clinical trials are warranted to validate its therapeutic potential.

### Emerging lines of research

In recent years, emerging lines of PVR research have increasingly focused on pathway-specific and regenerative strategies: inhibition of Rho-associated kinase (ROCK) has been proposed as an anti-fibrotic approach in vitreoretinal disease [88], and a multicenter clinical trial is currently evaluating topical netarsudil to prevent PVR after high-risk retinal detachment surgery [89].

Targeted therapies against key transcriptional and profibrotic drivers include small-molecule RUNX1 inhibition with the topical nanoemulsion Ro5-3335 and an mRNA-encoded dominant-negative RUNX1 “trap” that suppresses vitreoretinal disease in experimental models [90, 91], as well as modulation of connective tissue growth factor (CTGF), which promotes RPE EMT and ECM synthesis and can be targeted by intravitreal CTGF-neutralizing antibodies in experimental PVR [92, 93]. In parallel, regenerative and exosome-based concepts are evolving, with vitreous exosome proteomics and retinal disease models highlighting extracellular vesicles as mediators of tissue remodeling [94, 95] and exosomes derived from platelet-rich plasma (PRP-Exos) shown to activate YAP and enhance the fibrogenic activity of Müller cells, suggesting that autologous PRP-based exosome therapy could be harnessed or specifically modulated in future PVR-directed interventions [96].

## Discussion

To date, there is insufficient evidence supporting an effective non-surgical intervention for the prevention or treatment of PVR. As a result, established surgical procedures remain the only proven therapeutic option for manifest disease.

Clinical trials in vitreoretinal surgery, but also in all other surgical subspecialities, face methodological challenges that differ fundamentally from those encountered in pharmaceutical research. Standardizing complex surgical techniques, accounting for variation in surgeon experience, and managing urgent or emergency presentations make true randomization and blinding difficult, often resulting in underpowered studies with inherent bias [97]. Rapid advances in surgical technology and visualization further complicate trial design, as techniques considered state-of-the-art at trial initiation may already be outdated by the time recruitment is completed, limiting external validity. Resource constraints, due to the limited sponsorship typically available for surgical rather than industry-funded pharmaceutical trials, affect patient recruitment, systematic data monitoring, and long-term follow-up. Ethical considerations also play a critical role: given the time-sensitive nature of retinal detachment and PVR surgery, random assignment or delaying intervention is frequently unacceptable to clinicians and patients. Competition with concurrent pharmaceutical studies for eligible participants further complicates recruitment. Finally, publication bias toward positive findings leads to underreporting of neutral or negative trials, distorting the evidence base and complicating meta-analyses. Collectively, these factors help explain why promising preclinical treatments often fail to demonstrate consistent clinical efficacy and highlight the need for more robust and innovative trial designs in PVR research.

Until pharmacological strategies with a favorable safety profile become available, the use of experimental agents for primary prevention should be restricted to high-risk patients to enhance the statistical power and clinical relevance of future trials. Careful identification of risk factors is essential for consistent stratification [6, 26]. Although several major risk factors, such as ocular trauma, giant retinal tears, vitreous hemorrhage, or aphakia, are well recognized, additional, yet unidentified factors are likely to contribute to PVR susceptibility; therefore, continued investigation remains crucial.

Dosing considerations represent a major limitation in the current evidence base for adjunct pharmacological PVR therapies. For both MTX and corticosteroids, the heterogeneity of administered doses, injection frequencies, and timing relative to surgery significantly restricts comparability across studies. This variability complicates interpretation of treatment effects and prevents the establishment of standardized, evidence-based dosing regimens. In particular, MTX requires sustained exposure to achieve antiproliferative activity, yet the optimal therapeutic window and cumulative dose remain undefined. Similarly, for intravitreal corticosteroids, differences in formulation, release kinetics, and tamponade-dependent pharmacokinetics further limit meaningful cross-study comparison.

Given these challenges, sustained-release drug delivery systems represent a promising avenue for achieving stable, therapeutically relevant intraocular concentrations while avoiding concentration fluctuations inherent to repeated bolus injections. The early development of MTX-based sustained-release systems, including liposomal and implantable platforms, reflects a shift toward improving pharmacokinetic reliability and safety.

Multimodal strategies combining anti-inflammatory and antiproliferative agents are conceptually compelling, however the available clinical evidence shows that even combination treatments have so far not yielded sufficient efficacy in preventing PVR [27]. With advances in drug delivery, molecular targeting, and early intraoperative intervention, the rationale for revisiting combination approaches in future trials remains strong.

To advance the field, future clinical studies should also consider formal dose-escalation trials to define safe and effective dosing ranges. Beyond optimization of current agents, research is increasingly shifting toward targeted molecular interventions. Emerging approaches such as ROCK inhibitors, CTGF modulators, and RUNX1-targeted therapies aim to modulate specific profibrotic signaling pathways rather than relying solely on broad-spectrum antimetabolites. Notably, the first clinical trial evaluating ROCK inhibition for PVR prevention is expected to complete enrollment in 2025, underscoring the growing momentum behind mechanism-based treatment strategies.

## Data Availability

No datasets were generated or analyzed during the current study.
